# How Do Executive Functions Influence Children’s Reasoning About Counterintuitive Concepts in Mathematics and Science?

**DOI:** 10.1007/s41465-023-00271-0

**Published:** 2023-09-21

**Authors:** Iroise Dumontheil, Hannah R. Wilkinson, Emily K. Farran, Claire Smid, Roshni Modhvadia, Denis Mareschal, the UnLocke team, Derek Bell, Annie Brookman-Byrne, Andrea Gauthier, Roos de Jong, Wayne Holmes, Sveta Mayer, Su Morris, Kaśka Porayska-Pomsta, Dilini Sumanapala, Michael Thomas, Andy Tolmie

**Affiliations:** 1 Centre for Educational Neuroscience, Birkbeck, University of London, London, UK; 2 Centre for Brain and Cognitive Development, Birkbeck College, University of London, Henry Wellcome Building, Malet Street, London, WC1E 7HX UK; 3 School of Psychology, University of Surrey, Guildford, UK; 4 Department of Clinical, Educational and Health Psychology, University College London, London, UK

**Keywords:** Counterintuitive reasoning, Misconceptions, Executive function, Children, Mathematics, Science

## Abstract

Many scientific and mathematical concepts are counterintuitive because they conflict with misleading perceptual cues or incorrect naive theories that we build from our everyday experiences of the world. Executive functions (EFs) influence mathematics and science achievement, and inhibitory control (IC), in particular, might facilitate counterintuitive reasoning. Stop & Think (S&T) is a computerised learning activity that trains IC skills. It has been found effective in improving primary children’s mathematics and science academic performance in a large scale RCT trial (Palak et al., 2019; Wilkinson et al., *Journal of Cognitive Enhancement*, *4*, 296–314, 2020). The current study aimed to investigate the role of EFs and the moderating effects of S&T training on counterintuitive mathematics and science reasoning. A sample of 372 children in school Years 3﻿ (7- to 8-year-olds) and 5 (9- to 10-year-olds) were allocated to S&T, active control or teaching as usual conditions, and completed tasks assessing verbal and visuospatial working memory (WM), IC, IQ, and counterintuitive reasoning, before and after training. Cross-sectional associations between counterintuitive reasoning and EF were found in Year 5 children, with evidence of a specific role of verbal WM. The intervention benefited counterintuitive reasoning in Year 3 children only and EF measures were not found to predict which children would most benefit from the intervention. Combined with previous research, these results suggest that individual differences in EF play a lesser role in counterintuitive reasoning in younger children, while older children show a greater association between EFs and counterintuitive reasoning and are able to apply the strategies developed during the S&T training to mathematics and science subjects. This work contributes to understanding why specifically the S&T intervention is effective. This work was preregistered with the ISRCTN registry (TRN: 54726482) on 10/10/2017.

**Supplementary Information:**

The online version contains supplementary material available at 10.1007/s41465-023-00271-0.

## Introduction

Extensive research has found that executive functions such as inhibitory control and working memory are associated with academic performance in mathematics and science across childhood and adolescence (Cragg & Gilmore, 2014; Friso-Van den Bos et al., 2013; Meltzer, 2018; Tolmie et al., 2016). Efforts have been made to investigate which aspects of mathematics (e.g. fact retrieval, procedural skills, or conceptual reasoning) may be influenced by executive functions (EFs) (Cragg et al., 2017), and for both mathematics and science, the role EFs may play in overcoming misconceptions.

Indeed, many scientific and mathematical concepts are counterintuitive because they do not align with misleading perceptual cues and naive theories we build from our experiences of the world (Allen, 2014; Spooner, 2012). For example, a naïve theory in science is that larger organisms have larger cells than smaller organisms; it is counterintuitive that in fact larger organisms have more cells. Consequently, science and mathematics misconceptions develop and are routinely reinforced by our everyday experiences and beliefs, but are factually incorrect (Mareschal, 2016). While many early models of conceptual ‘change’ suggested that initial naïve theories are altered or replaced when conceptual learning occurs (e.g. Hewson, 1981; Nersessian, 1989; Villani, 1992), there is a growing body of evidence to suggest that these incorrect intuitive beliefs actually co-exist and are in conflict with correct scientific and mathematical representations (see Potvin et al., 2020 for review). Cognitive control of thought and behaviour is then required to overcome this conflict and inhibit interference from persistent misconceptions (Carey, 2000; Dunbar et al., 2007). As a consequence, in science and mathematics education, children often need to inhibit their pre-existing, intuitive beliefs, or their immediate perceptions, in order to correctly reason about academic concepts (Houde et al., 2000; Mareschal, 2016; Renouard & Mazabraud, 2018; Vosniadou et al., 2018). Drawing on the example used above, in science, children need to inhibit their intuitive reasoning that larger organisms have larger cells than smaller organisms, if they are to correctly learn that in fact larger organisms have more cells. Similarly, in mathematics, children must inhibit certain knowledge such as the relative size of integers (e.g. two is smaller than four) when comparing the relative size of fractions (e.g. ½ is larger than ¼).

On the basis of this evidence, we developed a computer-assisted learning activity (Stop & Think; S&T) that was designed to train primary school children (specifically, 7- to 10-year-olds) to use their inhibitory control (IC) skills when initially broaching a mathematics or science problem (Gauthier et al., 2022a, b). The training was based on two elements. First, children were told that in some mathematics and science problems, the first response that comes to mind is not necessarily the correct response. This aspect of the training was based on evidence that, when trying to improve adults’ performance on a counterintuitive logical reasoning task, explaining the logic for resolving the problem was not enough. In addition, it was necessary to include warning elements to the training (‘In this problem, the source of the error lies in a habit we all have of concentrating on…’, ‘… to not fall into the trap …’) (Houde et al., 2000). Second, children were encouraged to stop and think before giving their answers, and a delay was implemented before a response could be provided. This was based on research demonstrating that children perform better on tasks requiring IC when they are forced to delay responding. This is because a delay allows time for the prepotent response to dissipate and a more considered response to be formed (Diamond et al., 2002; Simpson & Riggs, 2007). Importantly, the IC training was embedded within mathematics and science problems which are typically considered to be counterintuitive to primary school children (e.g. Allen, 2014; Hansen et al., 2017; Pine et al., 2001; Ryan & Williams, 2007). This was based on evidence that training should be applied directly to the domain of interest, with the aim of strengthening content-specific neural connections (Botvinick & Cohen, 2014; Li et al., 2021). In other words, IC training likely needs to be embedded within subject-specific content, to allow children to appropriately apply these trained skills in the appropriate context.

A pilot study indicated that the S&T computerised learning activity could improve accuracy on a counterintuitive reasoning mathematics and science task in Year 3 (7- to 8-year-olds) but not Year 5 (9- to 10-year-olds) children (Wilkinson et al., 2020). There was also limited evidence (due to incomplete data) of higher attainment on standardised science tests for Year 3 children. The effectiveness of S&T was then evaluated through a large scale randomised controlled trial (RCT) including 6672 Years 3 and 5 children drawn from 89 schools across England (Palak et al., 2019). Classes were randomly assigned to one of three conditions: S&T, an active control consisting of a social skills training programme (‘See + ’) or Teaching as Usual (TAU; 25% of the sample). At the end of the training, half of the children completed a standardised mathematics test, the other half a standardised science test. Analyses controlled for an early measure of academic achievement (Early Years Foundation Stage Profile, collected at the end of Reception when children are 5 years old) and were stratified based on the number of classes schools had in each year. Primary analyses combined Years 3 and 5 children, and combined See + and TAU into a single Control group. Children in the intervention condition (S&T) performed significantly better than those in the Control group in science (Hedge’s *g* = 0.12, 95% CI (0.02, 0.22)), making the equivalent of two additional months’ progress. Children in S&T were also marginally significantly better in mathematics (Hedge’s *g* = 0.09, 95% CI (− 0.01, 0.19)), making the equivalent of one additional month’s progress. However, closer inspections revealed that S&T benefits were largely driven by the Year 5 children in the intervention condition, who tended to make more progress in mathematics (Hedge’s *g* = 0.14, 95% CI (− 0.002, 0.28)) and made significantly more progress in science (Hedge’s *g* = 0.17, 95% CI (0.03, 0.32)) than children in the Control (TAU and See + combined). Moreover, Year 5 pupils in the S&T condition made significantly greater progress in science and mathematics achievement compared to those in the active control (See +), suggesting that intervention benefits were not simply a result of participating in a novel computerised intervention. No significant differences were observed in Year 3 children.

These findings paint a puzzling picture of the effects of the inhibitory control intervention. On the one hand, the positive effects obtained in the RCT suggests that training inhibitory control within mathematics and science domains can help improve children’s academic performance. On the other hand, the nuanced pattern of results obtained between Year 3 and Year 5 pupils across studies raises questions about the mechanisms by which inhibitory control training impacts on academic performance at these different ages. The current study was planned, in parallel to the RCT, to investigate the mechanism of impact of the Stop & Think intervention by collecting a battery of cognitive measures on a smaller sample of children and exploring how IC and other EFs modulate the impact of S&T on children of different ages. Indeed, other aspects of cognitive control, in particular working memory (Brookman-Byrne et al., 2019; Bull & Lee, 2014; Cragg & Gilmore, 2014; Cragg et al., 2017; Donati et al., 2019; Gilmore et al., 2015; Khng & Lee, 2009; St Clair-Thompson & Gathercole, 2006) have been found to associate with general mathematics and science achievement. One study in adults found that working memory played a role in overcoming a salient intuition in a reasoning problem (Monty Hall Dilemma; De Neys & Verschueren, 2006), however, little research has tried to link working memory to counterintuitive mathematics and science reasoning specifically. Kwon, Lawson and colleagues found that adolescents with lower inhibitory control (measured by perseverative errors on the Wisconsin Card Sorting Test), planning or working memory, showed poorer scientific reasoning (Kwon & Lawson, 2000) and less benefit of individual tutoring in proportional reasoning (Kwon et al., 2000). In another study, spatial working memory and planning, but not response inhibition (Stop Signal task) associated with conceptual learning in biology (Rhodes et al., 2014) and chemistry (Rhodes et al., 2016) in 12- to 13-year-olds. As this research was on science topics and in adolescent participants, more research is needed to investigate the specificity of associations between EFs and math and science counterintuitive reasoning in childhood.

In what follows, we present detailed analyses of the pre-training association of a collection of cognitive measures (inhibitory control, verbal working memory, visuospatial working memory, vocabulary, and non-verbal reasoning) with accuracy on counterintuitive mathematics and science problems drawn from the primary school English national curriculum, and with improvements in performance associated with the S&T intervention. We focus on these types of problems because they are central to the proposal that one pathway through which IC acts to promote correct performance in mathematics and science is by inhibiting incorrect intuitive answers and allowing the valid answer to be selected. However, we acknowledge that IC has also been found to influence other aspects of mathematics, such as factual knowledge and procedural skills (Cragg et al., 2017). These detailed analyses in two separate school Year groups will allow us to answer questions about whether the effectiveness of the intervention is mediated by other executive functions or other participant variables.

Our hypotheses were that EF supports science and mathematics counterintuitive reasoning, that the S&T intervention can improve counterintuitive reasoning by encouraging children to use their IC skills in a science and mathematics context, and that individual differences in EF may influence the impact of S&T. An understanding of the extent to which EF training, when embedded within a science and maths context, transfers to science and maths performance more broadly will provide both practical and theoretical insight to our understanding of the mechanisms that support science and mathematics performance in children.

In exploratory analyses, we predicted that, before the intervention, science and mathematics counterintuitive reasoning would be associated cross-sectionally with EF measures (inhibitory control, verbal working memory, visuospatial working memory). In preregistered analyses, we further predicted that, as a result of the intervention, children would show improved performance on science and mathematics counterintuitive reasoning (preregistered hypothesis 1a, 10.1186/ISRCTN54726482), and that children may also show far transfer, demonstrated by domain general improvements in inhibitory control (but not working memory) (preregistered hypothesis 3a). While we originally predicted improvements in science and mathematics academic achievement (preregistered hypothesis 1b) the data were only available at T2, and for a reduced sample (as half of the participants completed the one-hour-long standardised mathematics test, and half the science test). As this had been already demonstrated in the S&T RCT (Palak et al., 2019), this was not investigated further in the current study. Finally, exploratory analyses investigated whether individual differences in pre-training performance, EFs or IQ, may influence the impact of the S&T intervention.

## Material and Methods

### Participants

A sample of 372 children from 21 schools in England took part in this study. Two age groups, 7- to 8-year-olds (Year 3) and 9- to 10-year-olds (Year 5), were chosen for this study and were analysed separately to match the analytic approach adopted in the S&T RCT (Palak et al., 2019). The Department for Education (2018) records were used to obtain the percentage of pupils with free school meals (FSM) for each school. FSM is an indirect index of socio-economic status. The proportion of children with FSM ranged from 0.6 to 30.6% across schools (*M* = 12.36; *SD* = 7.99), with eight of the 21 schools having a larger proportion of pupils with FSM than the national average of 13.6%.

We aimed to recruit 180 children for this study. We initially recruited 159 children (from 15 schools) in a first wave of data collection (which took place in October–November 2017 for time 1 (T1) and in February–March 2018 for time 2 (T2)) that was embedded within a larger RCT study (Palak et al., 2019). However, because of timing and other constraints arising from the primary RCT study, our sample was very uneven with regards to conditions. We therefore set-up a second wave of data collection (prior to looking at the collected data in hand) in March (T1) and June-July (T2) 2018 in six additional schools, resulting in the recruitment of an additional 213 participants. For the first wave, randomisation of classes to each intervention condition was implemented by an external evaluator for the RCT study. For the second wave, allocation of classes to the intervention conditions was pseudo randomised with the constraint that the number of pupils taking part in each class balanced the number of participants in each intervention condition and Year group across the joint first and second wave samples of the current study.

Parents and children could opt-out of the interventions. Parental informed opt-in consent was obtained for all assessments. While there was no exclusion criterion for participation in the interventions, consent forms for the assessments stipulated that children must have no known developmental or neurological disorder to participate. Finally, the study was approved by the Birkbeck Department of Psychological Sciences Research Ethics Committee.

Two participants were excluded from all analyses; one was an outlier (further than 3.29 *SD* from the mean) on age and one was an outlier on IQ. The final sample for cross-sectional T1 analyses included 187 Year 3 children (7.17–8.52 years, *M* = 7.87, *SD* = 0.33; 52.4% males) and 183 Year 5 children (8.97–10.56 years, *M* = 9.90, *SD* = 0.36; 57.4% males) (see Supplementary Table S1 for the *n*’s for each measure at T1). Table 1 provides *n*’s and descriptive statistics for each Year group and condition included the longitudinal analyses of science and mathematics counterintuitive reasoning (see Supplementary Materials C for comparison of the S&T and Control groups on demographic variables). Presentation of these values for the separate control conditions can be found in Supporting Materials D and Table S4. Note that participant numbers varied by analyses due to an incomplete test battery for some pupils at one or both time points.

**Table 1:** Descriptive statistics of the samples included in the analyses of the effect of the Stop & Think (S&T) intervention on measures of science and mathematics counterintuitive reasoning

Year group	Condition	*N*	Age at T2, *M* (*SD*)	% Males	IQ, *M* (*SD*)
Year 3	Stop & Think	58	8.28 (0.29)	62.1	100.9 (15.4)^a^
	Control	111	8.15 (0.32)	49.5	103.3 (14.6)
Year 5	Stop & Think	93	10.14 (0.33)	62.4	104.2 (15.6)^a^
	Control	77	10.35 (0.32)	50.6	101.9 (12.2)

^a^One IQ value was missing for this group

### Procedure

**Fig. 1:**
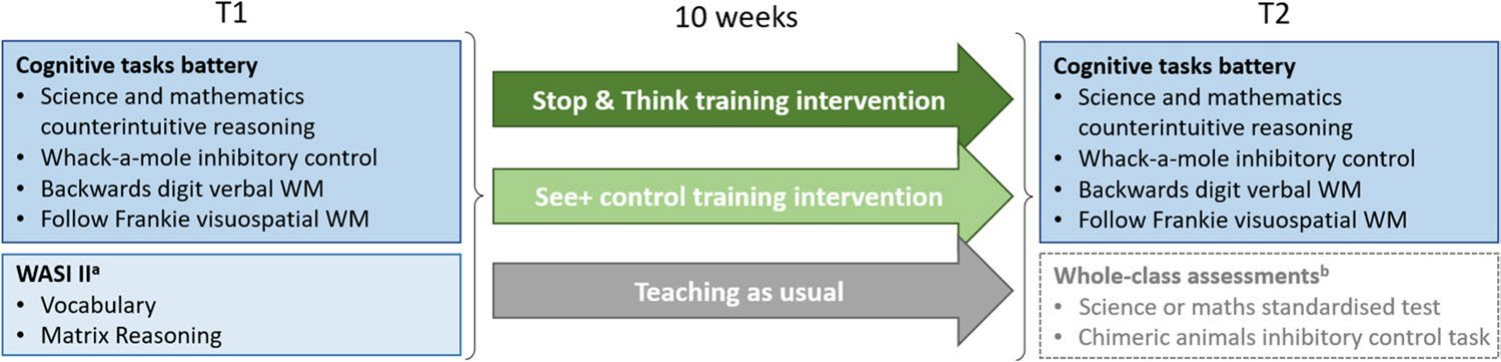
Illustration of the procedure and data collection for this study. ^a^The WASI II sub-tests were re-administered at T2 for 47 participants because of an error of test administration at T1. ^b^These measures were not analysed as part of the current study

A battery of tasks assessing EF skills and counterintuitive reasoning in science and mathematics was administered at both time points. In addition, children completed the WASI-II subtests at T1 (repeated at T2 if T1 administration was incorrect, *n* = 47). Children also performed the *Progress Tests in Mathematics* and *Progress in Science* (GL assessment, 2015a, b, c, d) and a chimeric animals inhibitory control task at T2 only, administered either to the whole class or to small groups. These measures were not analysed as part of the current study. Figure 1 summarises the timings of data collection.

Participants were tested individually on the behavioural battery in a quiet space at school, outside the classroom. In some cases, the battery was split across two days due to time constraints at the schools. At T1, up to an hour was allocated for all tasks; at T2, most children completed all tasks within half an hour. Tasks were not administered in a specific order to allow testers to administer tasks at the most suitable times (e.g. the backwards digit task when the area was quietest, or shorter tasks when a school break was due). Children were tested on Acer Swift 3 laptops, intel core i3, 7th gen. The screen resolution was 1280 × 1024 and speaker volume and cursor speed were both set to 50%. Identical headphones and mice were used with all participants.

Testers introduced themselves as scientists from the Centre for Brain and Cognitive Development, explaining that they were conducting a science project looking at how children think when solving problems. Participants were asked if they were happy to take part and were assured that they could have a break or stop at any point. Testers followed the on-screen instructions for the administration of all behavioural tasks and were blind to the experimental conditions that the classes had been allocated to. Participants were given small prizes for taking part.

### Conditions

#### Stop & Think (S&T)

**Fig. 2:**
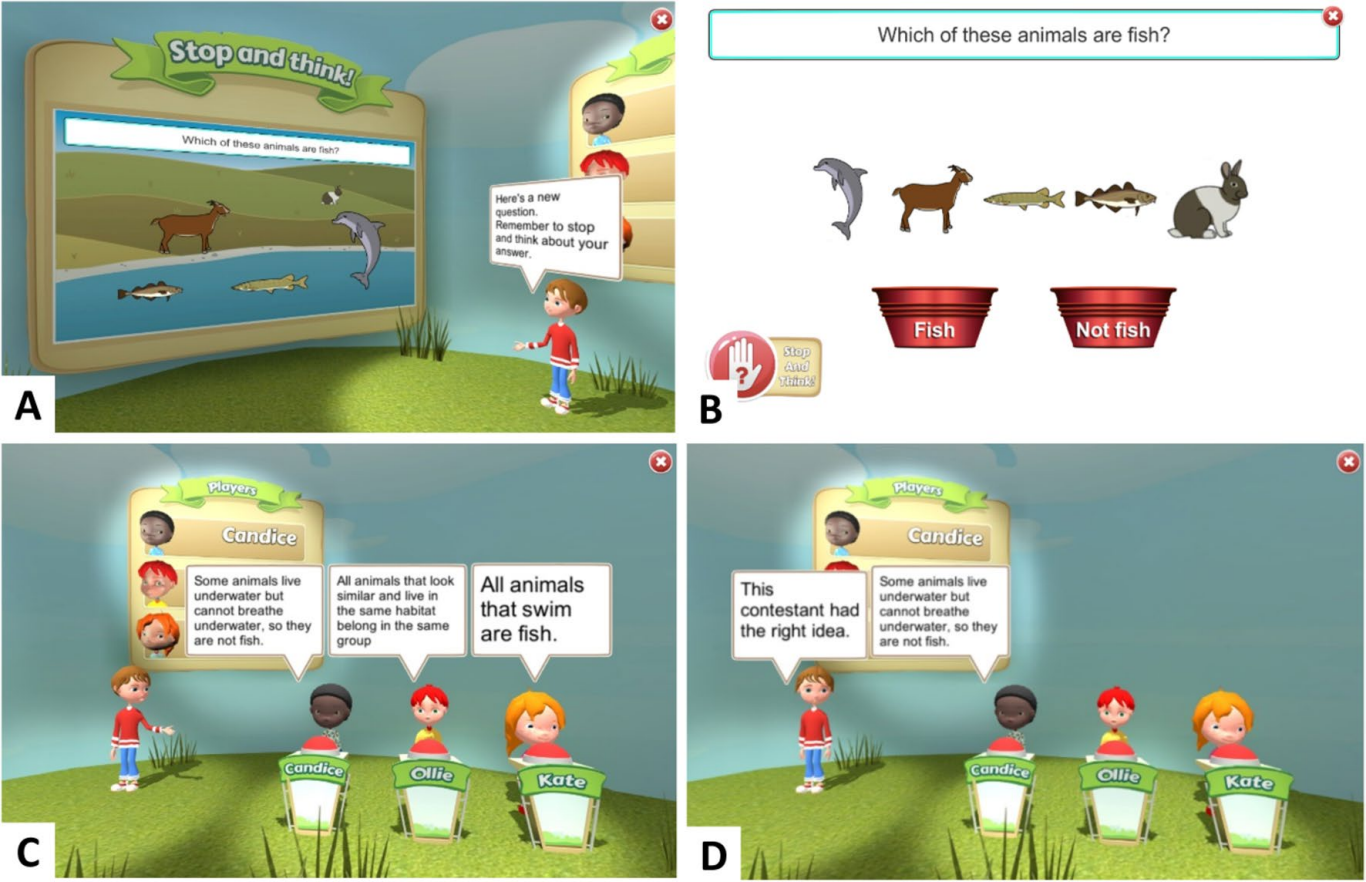
‘Stop & Think’ interface and inhibitory control prompts. **A** Game show host Andy reminding the user to ‘stop and think’ before responding to the task. **B** Pulsing ‘Stop and Think’ logo (bottom left of screen). **C** Contestants’ presenting their thoughts (reasoning) about the task. In this example, the character Candice has the correct reasoning, Ollie has the misconception, and Kate is more generally incorrect. **D** The contestant with the correct reasoning is revealed

S&T is a computer-based intervention that was developed to address the learning of counterintuitive concepts in Year 3 and Year 5 children by embedding IC training within science and mathematics content and lessons. The intervention encourages children to repeatedly practise inhibiting their intuitive response in favour of a delayed and more considered response, i.e. to ‘stop and think’, while solving age-relevant counterintuitive science and mathematics problems. A technology-enhanced learning approach (Goodyear & Retalis, 2010) is used to deliver IC training in a virtual game-show format, in which an animated character named Andy presents science and mathematics problems to the user and three virtual gameshow contestants (Fig. 2).

A short introductory video first explains, with examples: that some concepts are counterintuitive; how this can lead to mistakes in science and mathematics learning; and how these mistakes might be avoided, i.e. through the use of IC. Throughout the intervention, prompts to ‘stop and think’ are used to encourage children to use their IC when solving science and mathematics problems. Before presenting each problem, Andy reminds the user to ‘stop and think’. This is followed by a 5-s pulsing S&T logo, during which time the response screen is visible but locked, forcing the user to withhold their prepotent response and encouraging them to think about the question before responding (Fig. 2B).

The three virtual contestants model ‘stopping and thinking’ while reasoning about science and mathematics problems (Fig. 2 C and D). This was informed by research which demonstrates the benefit of collaborative learning, including the use of virtual characters as learning peers (e.g. Porayska-Pomsta et al., 2013, 2018). Through educational tools, such as Think-Pair-Share, Concept Cartoons, and ScotSPRinG, previous research has demonstrated that children can enhance their learning by comparing their own beliefs with those of others and by reflecting on explanations to problems in relation to their own thinking (Dabell et al., 2008; Keogh & Naylor, 1999; McTighe et al., 1988; Naylor & Keogh, 2013; Tolmie, 2014). In S&T, the virtual contestants are shown presenting their thoughts on the current science or mathematics problem. One contestant presents the correct line of reasoning, one holds an incorrect intuitive belief, i.e. a misconception, and the third is more generally incorrect or states ‘I don’t know’. The contestants’ reasoning is presented adaptively after two incorrect attempts or immediately after a correct response. This format was intended to encourage children to consider the contestants’ reasoning before they make a third attempt (to help develop their own reasoning), or after they provide the correct response (to reflect upon why their answer was correct).

**Fig. 3:**
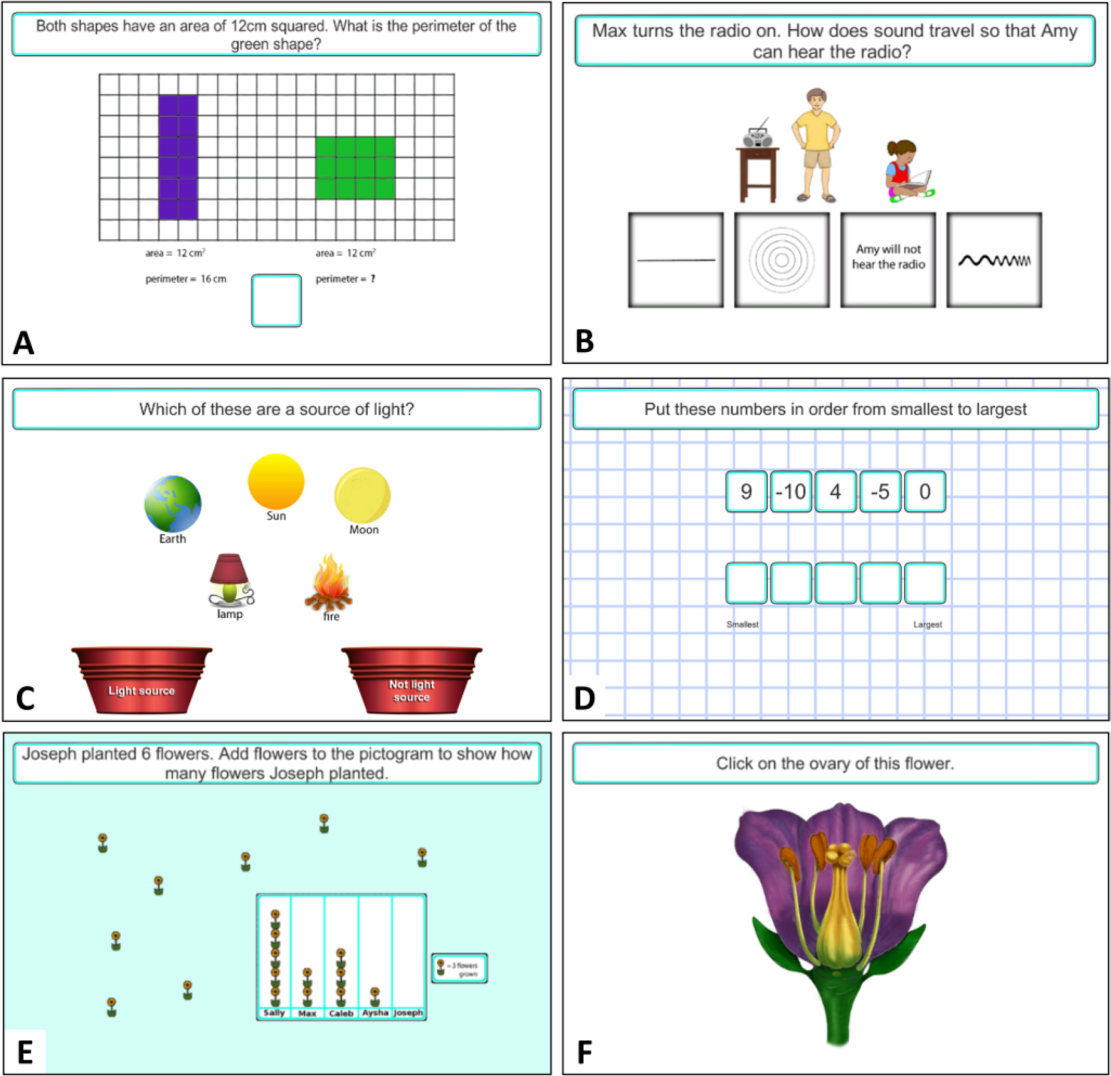
Stop & Think problems examples with different response types

The science and mathematics questions were developed by compiling a set of problems based on common misconceptions that were age-appropriate for the National Curriculum in England (Allen, 2014; Cockburn & Littler, 2008; Department for Education, 2013a, b; Gates, 2002; Hansen et al., 2017; Pine et al., 2001; Ryan & Williams, 2007). Questions were reviewed by teachers to check their appropriateness. Sessions were delivered in a fixed order which progressed from relatively easy concepts (based on the curriculum from the previous academic year) to more challenging concepts (based on the curriculum of the current academic year) to allow children to first practise using the ‘stop and think’ skill with familiar content, before moving on to apply this IC skill to more difficult concepts. Each session includes one mathematics and one science concept; their order is pseudo-randomised. For each concept, the user is first presented with an ‘Exploratory’ problem (Fig. 2) which allows multiple response attempts, with progressively greater levels of support offered each time an incorrect response is given. This is followed by up to five ‘Structured Practice’ problems based on the same science or mathematics concept, which provide further opportunities to practise the ‘stop and think’ skill at increasing levels of difficulty, with different questions and stimuli, and with varied response formats (Fig. 3). Previous research has suggested that training with variable and adaptively more complex tasks can help keep the user motivated and lead to greater generalisation of trained skills to real-world situations (Green & Bavelier, 2008; Klingberg, 2010; Morrison & Chein, 2011). When all problems for the session are complete, or 12 min has passed since logging in, the session automatically ends.

Rather than providing additional science and mathematics content to teaching as usual, S&T replaced the first 12 min of science or mathematics lessons three times a week for 10 weeks (maximum dose of 360 min). In other words, pupils in the S&T condition did not get any additional content or additional exposure time to mathematics and science material. The teacher led the sessions as a whole-class activity using the interactive whiteboard. Teachers were instructed to enter the class response into the software following either a class vote or an individual child volunteering their response. The teacher’s role was to guide the class through the problems and keep pupils on-task, rather than to offer teaching of science or mathematics content or prompt pupils towards the correct response.

#### Active Control (See +)

See + (Social Emotional Engagement through observation) was developed as the active control for S&T. The two interventions were designed to be matched in terms of the novelty of using a technology-enhanced activity in the classroom, including the same virtual characters. The interventions were also matched in duration and frequency of sessions and teacher involvement in whole-class delivery. Importantly, See + does not involve any IC training, counterintuitive reasoning, or science and mathematics curricula content, but instead targets the domain of socio-emotional cognition (Nader-Grosbois & Day, 2011). Furthermore, See + was not delivered during mathematics and science lessons, but instead was delivered at a time in the school day normally dedicated to Personal, Social, Citizenship and Health Education (PSCHE), when children would ordinarily be engaging in a social and emotional skills curriculum.

See + sessions follow three phases of engagement. First, the users observe a short animation in which virtual characters take on different roles in a social scenario. Then, they are presented with a multiple-choice question regarding the actions of these characters. Next, children are required to reflect on the beliefs and emotions of the characters by selecting the most appropriate response from images of emotional expressions, written statements of a character’s thoughts, or by manipulating a rating scale that morphs the character’s emotional expression. Finally, children are encouraged to think of appropriate resolutions to the social dilemmas through a class discussion.

#### Teaching as usual (TAU)

Participants in TAU were not exposed to S&T or See + . The allocation of conditions was such that, in each school, the S&T condition was only implemented in one Year group (and all classes within that Year group) (e.g. Year 5), with TAU or See + in the other Year group (e.g. Year 3), to reduce contamination bias across teachers or pupils in participating schools with more than one class per school Year.

### Measures

#### Wechsler Abbreviated Scale of Intelligence II

The Vocabulary and Matrix Reasoning subtests of the WASI-II (Wechsler, 2011) were administered at T1 to assess the participants’ IQ. Forty-seven children had the WASI-II re-administered at T2 as their vocabulary subtests were incorrectly administered at T1 (one tester incorrectly stopped the test after two, rather than three, incorrect responses). Data collected from repeated administration of the WASI II twice within 12 to 88 days interval have shown there was acceptable (0.79) to excellent (0.90) test–retest stability coefficients for the subtests (i.e. the separate Vocabulary and Matrix Reasoning measures) and good (0.87) to excellent (0.95) coefficients for the composites (i.e. the combined IQ measure) (McCrimmon & Smith, 2013).

#### Science and Mathematics Counterintuitive Reasoning Task

**Fig. 4:**
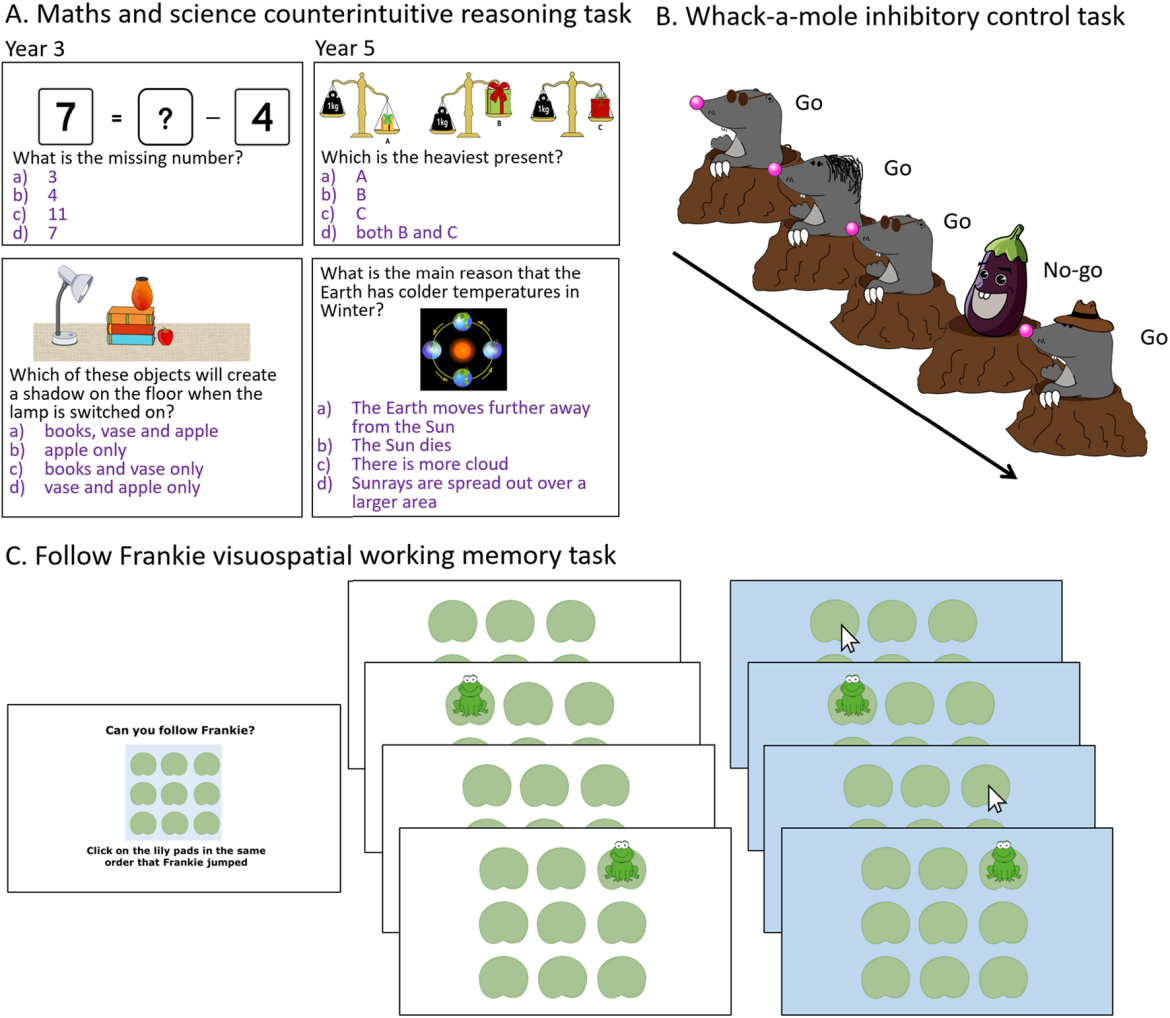
Cognitive task battery. **A** Example stimuli from the science and mathematics counterintuitive reasoning task for Year 3 (left) and Year 5 (right) children. **B** Example sequence of stimuli for the whack-a-mole inhibitory control task. Children were asked to press the spacebar when they saw a mole (go trial) but to inhibit their response when they saw an aubergine (no-go trial). **C** Example trial of the follow Frankie visuospatial working memory task. Children saw Frankie jump on lily pads (left, white background) and were asked to click on the lily pads with their mouse to repeat the sequence (right, blue background)

A novel science and mathematics counterintuitive reasoning multiple-choice task was administered at T1 and T2. Years 3 and 5 children were given different age-appropriate sets of 28 questions (14 in science, 14 in mathematics) in-line with the National Curriculum. Within each subject, 12 questions were based on counterintuitive concepts (Fig. 4A) and two on concepts that were not counterintuitive (not included in the analyses, but used to prevent participants from thinking that their intuitive response is always incorrect). Eight of the counterintuitive questions were based on concepts covered in the S&T intervention and four were based on novel concepts. Each item had four alternative forced choice response options and participants were required to respond using keys labelled ‘a’ to ‘d’ on the testing laptops with their preferred index finger. One response option was correct and of the three incorrect response options, one option was the misconception response (i.e. an intuitive, but incorrect response). An audio recording of each question was played via headphones. Participants were given a maximum time of 32 s to respond. After 25 s had elapsed, the text ‘choose one!’ was displayed to encourage participants to respond before the next question. Participants were told that they would have a fixed time to answer each question but that they should try to respond as accurately as they could and to make a best guess if they were unsure. There was no practice trial, but participants were given the opportunity to ask the tester to explain the instructions further. The measure of performance on this task was accuracy on the counterintuitive reasoning trials. Science and mathematics trials were combined to increase the number of trials entered in the analyses and because we are interested in cognitive processes supporting counterintuitive reasoning that are common across subjects. Pearson correlations indicate that counterintuitive reasoning accuracy in mathematics and science were mostly significantly positively correlated (Year 3: T1, *r* = 0.142, *p* = 0.054; T2, *r* = 0.159, *p* = 0.037; Year 5: T1, *r* = 0.427, *p* < 0.001; T2, *r* = 0.528, *p* < 0.001).

#### Inhibitory Control

We measured inhibitory control using a version of the computerised ‘whack-a-mole’ (WAM) go/no-go task (Shapiro et al., 2013). Participants were required to press the spacebar to respond to the go stimuli (moles) and to withhold from responding to no-go stimuli (aubergines) (Fig. 4B). Stimuli were shown until participants responded or for a maximum of 0.5 s. Feedback was shown in the form of cartoon-like ‘pow!’ for correct hits and ‘oops!’ for incorrect hits. Trials were separated by 1 s. A practice of four trials with one no-go trial was repeated if participants made one or more errors. The test phase had 75 go trials and 25 no-go trials. Performance was reported using a *d’* sensitivity index (Hautus, 1995; Stanislaw & Todorov, 1999) as well as mean reaction time (RT) for go trials (Shapiro et al., 2013). The *d*’ was chosen rather than No Go accuracy because there was a considerable range in Go accuracy (range Year 3 [63–100%], Year 5 [73–100%]) and *d’* includes both hits and false alarms. Children also performed a Flanker task but because of poor correlation between T1 and T2 performance, suggesting low reliability of the interference measure, these data were not included in the current study. Reliability of the included measures is presented below.

#### Working Memory

Verbal WM was assessed using the backwards digit task. The tester read aloud a series of numbers, which participants were asked to repeat back in reverse order. A practice trial included two digits and was repeated until the participant understood the task. Testing started with a sequence of three numbers (a span of 3). Visuospatial WM was assessed using an adaptation of the follow Frankie task developed by Morris and colleagues (Morris et al., 2019). Participants were required to remember and repeat a spatial sequence depicted using a frog jumping on an array of nine lily-pad location points arranged in a 3 by 3 array (Fig. 4C). Participants were shown an example sequence and example correct response. Children were encouraged to use the mouse, but if they found this difficult, they could respond using the laptop trackpad instead. The game began with a practice session involving three sequences of two jumps, which was repeated if children made two or more errors. Testing started with a span sequence of two. For both tasks, the span increased by one after four trials were completed if participants made fewer than two errors out of the four trials. The maximum span was seven. The final verbal WM and visuospatial WM scores were the total number of sequences correctly repeated. The same sets of number sequences were used at both testing time points.

### Data Analysis

Participants were considered outliers if their cognitive scores were more than 3.29 *SD* from the mean score across Year groups for cognitive tasks (same tasks for both year groups) and within Year group for science and mathematics tasks (different content for each year group). For T1 data, four participants were low performance outliers on the WAM task *d*’ measure and were excluded from analyses that included this task. At T2, seven participants (one of which was also a T1 outlier) were low performance outliers on the WAM task *d*’ measure and were excluded from intervention analyses that included this task (i.e. 10 participants in total). There were no other outliers.

#### Reliability of Measures

Pearson correlations were run between timepoints to obtain an estimate of the reliability of the measures and whether this differed between Year groups. Correlations were significant and ranged between 0.391 and 0.745 (Table 2). Correlations were similar in Years 3 and 5 for the inhibitory and visuospatial WM measures, but marginally higher for Year 5 than Year 3 for the verbal WM measure (*p* = 0.057) and significantly higher for Year 5 than Year 3 for the counterintuitive reasoning accuracy measure (*p* < 0.001) (Table 2).

**Table 2:** Correlation between T1 and T2 measures of executive function and counterintuitive reasoning

	T1 T2 Pearson correlation (*r*)		Fisher Z comparison (*Z*)
	Year 3	Year 5	Y3 vs. Y5
Verbal working memory score	0.452*** (*n* = 157)	0.606*** (*n* = 162)	− 1.905
Visuospatial working memory score	0.391*** (*n* = 179)	0.322*** (*n* = 172)	0.734
Inhibitory control *d*'	0.532*** (*n* = 176)	0.588*** (*n* = 167)	− 0.749
Inhibitory control go RT	0.488*** (*n* = 176)	0.435*** (*n* = 167)	0.618
Counterintuitive reasoning accuracy	0.526*** (*n* = 169)	0.745*** (*n* = 170)	− 3.440***

*** *p* < 0.001

#### T1 Cross-sectional Analyses

Cross-sectional analyses were not preregistered and were exploratory. The aim of these analyses was to demonstrate that science and mathematics counterintuitive reasoning is associated with executive function (inhibitory control, verbal working memory, visuospatial working memory) in children. Comparisons of the two year groups on IQ, verbal WM and visuospatial WM scores, and WAM *d’* are reported in Table S1. The key analyses were performed in three steps, separately for Year 3 and Year 5 participants. First, partial correlations controlling for age were run to assess associations between performance on the counterintuitive reasoning task and IQ and EF measures. Significant associations with EF were followed up through multiple regressions, where age was entered in a first step and EF measures in the second step, to (i) assess the variance explained by EF measures overall, and (ii) investigate whether individual EFs showed specific associations with counterintuitive reasoning performance. Finally, further multiple regressions were carried out in which IQ was entered as an additional regressor in the first step, to assess whether EF explained any variance in counterintuitive reasoning over and beyond IQ. Analyses considering vocabulary and matrix reasoning measures separately are reported in Supporting Materials B.

#### Intervention Analyses

Analyses of the main effects of the intervention were preregistered. The Control condition refers to TAU and See + combined (see Supplementary Materials D for pairwise comparisons of the three conditions). Intervention effects were tested using 2 (Time: T1, T2) × 2 (Condition: S&T, Control) mixed repeated measures analyses of variance (ANOVAs) using as dependent variables: (1) counterintuitive reasoning accuracy (i.e. the percentage of correct responses on counterintuitive items), (2) verbal WM score, (3) visuospatial WM score, and (4) whack-a-mole *d*’. Significant time by condition interactions were followed-up with simple main effects. In addition, as we had predictions for both significant and null results, Bayesian ANOVAs were performed post hoc for the key time × condition interactions using JASP (JASP Team, 2019). To quantify uncertainty about effect size and to obtain evidence in favour of a null hypothesis (Wagenmakers et al., 2018), we distinguished between experimental insensitivity (Bayes factor [BF] 10 and BF 01 < 3) and robust support for the alternative hypothesis (BF 10 > 3) or null hypothesis (BF 01 > 3) (Dienes, 2014). 

Exploratory analyses investigated predictors of improvements in counterintuitive reasoning. Multiple regressions were run to assess predictors of T2 counterintuitive reasoning accuracy, and in particular whether any predictor specifically explained variance in accuracy changes in the Stop & Think group compared to the control group.

#### Deviation from Pre-registration

All hypotheses from our large suite of measures collected in our broader project formed a single preregistration (10.1186/ISRCTN54726482). The current study addressed hypotheses 1a and 3a of our pre-registered plan (hypotheses 2 and 3b relate to neural imaging data and will form a separate paper). Hypothesis 1b relates to achievement data which was better analysed within the context of the whole RCT study due to limited sample size and lack of longitudinal data (see Palak et al., 2019). The current analyses therefore focused on the counterintuitive reasoning task data, and predictors of performance at T1 and of improvements through S&T training. The study deviated from our pre-registered plan for data collection and analyses as follows. First, as mentioned above, a second wave of data collection was carried out to increase the N overall but also the minimum N in each participant group. Second, intervention effects were assessed on all three measures of EF rather than IC only. Whilst we had predicted the intervention may show transfer to IC only, it is important to demonstrate that predicted null effects are supported statistically. For these additional analyses, we had predicted no significant intervention effect on these measures. Finally, we had planned on looking at both accuracy and RT measures of counterintuitive reasoning, but preliminary analyses indicated that RT data had low T1-T2 correlation (*r* < 0.1), we therefore did not include this measure in the analyses. Finally, we carried out additional analyses comparing effects between Year groups. Fisher r-to-z transformation was used to compare correlations, and Year group was included as a factor or interaction term in the ANOVAs or regressions.

## Results

### Time 1 Cross-sectional Analyses

Partial correlations controlling for age showed that EF and IQ were positively correlated to each other, except for verbal WM and IC in Year 3. Correlations were broadly stronger in Year 5 than in Year 3. Counterintuitive reasoning accuracy was associated with IQ and all EF measures in Year 5, but only with IQ in Year 3 (Table 3, see results with vocabulary and matrix reasoning measures in Supplementary Table S2). Fisher r-to-z transformation comparison of correlations between Years indicated there was a significant difference between Years for the correlations between counterintuitive reasoning accuracy and IQ (*Z*_Y3vsY5_ = -2.967, *p* = 0.003) and between counterintuitive reasoning accuracy and verbal WM (*Z*_Y3vsY5_ =  − 2.681, *p* = 0.007; all other -1.338 < *Z*’s <  − 0.402, *p’*s > 0.18). Partial correlations with the mean go RT measure of inhibitory control were not significant, except with the *d*' measure of the same task in Year 5 (see Supplementary Table S2), and were not analysed further.

**Table 3:** Partial parametric correlations between IQ, T1 executive function measures and mathematics and science counterintuitive reasoning accuracy, covarying age. N for each test is provided below the diagonal

Year	Measures	1	2	3	4	5
Year 3	1. IQ		0.292***	0.318***	0.161***	0.402***
	2. T1 verbal working memory	166		0.226**	0.124	0.064
	3. T1 visuospatial working memory	184	169		0.188*	0.120
	4. T1 inhibitory control	182	167	185		0.076
	5. T1 counterintuitive reasoning accuracy	180	165	183	181	
Year 5	1. IQ		0.332***	0.362***	0.242**	0.630***
	2. T1 verbal working memory	169		0.285***	0.226**	0.345***
	3. T1 visuospatial working memory	180	170		0.296***	0.256***
	4. T1 inhibitory control	178	168	180		0.179**
	5. T1 counterintuitive reasoning accuracy	180	170	182	180	

^†^*p* ≤ 0.10, *p ≤ 0.05, **p ≤ 0.01, ***p ≤ 0.001

Follow-up regression analyses assessed the specificity of the associations observed in Year 5. Out of the three EF measures only verbal WM explained unique variance in counterintuitive reasoning accuracy (Table 4, model A), and remained significant when IQ was included (model B). Visuospatial WM and IC did not predict unique variance in counterintuitive reasoning. While as a whole the EF measures explained 13.2% variance after controlling for age (model A), the EF measures did not explain any significant variance beyond age and IQ (model B).

**Table 4:** Follow-up multiple regression analysis of mathematics and science counterintuitive reasoning accuracy in Year 5 children

	Variables	Model A		Model B	
		β	*p*	β	*p*
(Step 1)	T1 age	0.046	0.619	0.060	0.344
	IQ			**0.544**	** < 0.001**
(Step 2)	T1 verbal WM	**0.274**	** < 0.001**	**0.151**	**0.026**
	T1 visuospatial WM	0.104	0.181	-0.025	0.712
	T1 inhibitory control	0.108	0.165	0.032	0.631
		Step 1:* R*^2^ = 0.2%, *n.s* **Step 2: Δ*R***^**2**^** = 13.2%, *p* < 0.001**		**Step 1:* R***^**2**^** = 35.1%,* p* < 0.001** Step 2: Δ*R*^2^ = 2.2%, *n.s*	

Parameter estimates and *p* values are provided for the final models. Significant effects are highlighted in bold

*WM* working memory

In Year 3, a similar multiple regression analysis showed that the three EF measures combined did not explain significantly more variance in counterintuitive reasoning accuracy than age alone (Δ*R*^2^ = 2.1%, *p* = 0.346). To test whether each EF predictor significantly differed between Year groups, the following formula was used: Z_Y3vsY5_ = (β_Y3_ − β_Y5_)/sqrt(SEβ_Y3_^2^ + SEβ_Y3_^2^)(Clogg et al., 1995). In line with the comparison of partial correlations, these comparisons indicated that verbal WM was a greater predictor of counterintuitive accuracy in Year 5 than Year 3 (*Z*_Y3vsY5_ =  − 2.029, *p* = 0.042), while visuospatial WM and inhibitory control standardised coefficients did not differ between Years (*Z*’s >  − 0.370, *p*’s > 0.7).

#### Intervention Effect on Counterintuitive Reasoning and Executive Functions

Means and standard deviations of counterintuitive reasoning task and executive function tasks performance at T1 and T2 are presented in Table 5. We examined whether changes from T1 to T2 in counterintuitive reasoning accuracy and changes in the three executive functions measures were significantly different in the S&T group compared to the Control group using 2 (time; T1, T2) × 2 (condition; S&T, Control) mixed ANOVAs. The predictions were of greater improvement in accuracy in counterintuitive task performance in the S&T group compared to the Control group, and of a possible increase in standard measures of inhibitory control. Results are presented in Table 6.

**Table 5:** Means and standard deviations (in parentheses) of counterintuitive reasoning accuracy and the three executive function measures, for Year 3 and Year 5 children at time 1 (T1) and time 2 (T2), split by condition: Stop & Think, Control

	Year 3						Year 5					
	Stop & Think			Control			Stop & Think			Control		
	*N*	T1 *M* (*SD*)	T2 *M* (*SD*)	*N*	T1 *M* (*SD*)	T2 *M* (*SD*)	*N*	T1 *M* (*SD*)	T2 *M* (*SD*)	*N*	T1 *M* (*SD*)	T2 *M* (*SD*)
Counterintuitive reasoning acc. (%)	58	32.5 (11.6)	44.8 (13.0)	111	35.5 (11.6)	40.5 (12.4)	93	45.5 (16.9)	51.0 (17.4)	77	42.9 (14.4)	47.8 (17.1)
Verbal working memory score	52	4.6 (1.8)	5.3 (2.6)	105	4.1 (2.4)	4.8 (2.5)	93	7.1 (2.7)	7.3 (3.3)	69	6.0 (2.5)	7.0 (2.7)
Visuospatial working memory score	59	8.8 (3.7)	9.2 (4.1)	120	9.1 (3.7)	9.8 (3.4)	97	11.9 (3.5)	12.0 (4.0)	75	11.8 (3.7)	12.6 (3.5)
Inhibitory control *d*’	56	2.08 (0.68)	2.37 (0.67)	120	1.92 (0.67)	2.22 (0.74)	93	2.62 (0.78)	2.80 (0.78)	74	2.57 (0.65)	2.8 (0.65)

The Control condition comprises the teaching as usual and See + conditions combined

**Table 6:** Results of 2 (time: time 1, time 2) × 2 (condition: Stop & Think, Control) mixed ANOVAs carried out on counterintuitive reasoning accuracy and the three executive function measures, for Year 3 and Year 5 children

	df	Main effect of time			Main effect of condition			Time × condition interaction			Bayesian statistics	
		*F*	*p*	η_p_^2^	*F*	*p*	η_p_^2^	*F*	*p*	η_p_^2^	BF01	BF10
Year 3												
Counterintuitive reasoning acc	**1, 167**	**86.5**	** < 0.001**	**0.341**	0.1	0.743	0.001	**15.4**	** < 0.001**	**0.084**	0.006	**147.403**
Verbal working memory	**1, 155**	**10.3**	**0.002**	**0.062**	2.4	0.120	0.016	< 0.1	0.905	< 0.001	**5.525**	0.181
Visuospatial working memory	1, 177	3.1	0.080	0.017	0.7	0.395	0.001	0.3	0.615	0.001	**5.618**	0.178
Inhibitory control	**1, 174**	**29.0**	** < 0.001**	**0.143**	2.6	0.110	0.015	< 0.1	0.901	< 0.001	**5.525**	0.181
Year 5												
Counterintuitive reasoning acc	**1, 168**	**32.5**	** < 0.001**	**0.162**	1.5	0.220	0.009	0.1	0.773	< 0.001	**5.952**	0.168
Verbal working memory	**1, 160**	**9.4**	**0.002**	**0.056**	3.3	0.071	0.020	3.6	0.060	0.022	1.120	0.893
Visuospatial working memory	1, 170	1.4	0.238	0.008	0.3	0.618	0.001	1.0	0.312	0.006	**3.663**	0.273
Inhibitory control	**1, 165**	**16.7**	** < 0.001**	**0.092**	1.0	0.822	< 0.001	0.3	0.589	0.002	**5.556**	0.180

The Control condition comprises the teaching as usual and See + conditions combined. Significant effects are highlighted in bold. Bayesian statistics are reported comparing a model including the time × condition interaction to a model including the two main effects only. BF01: evidence for the null hypothesis; BF10: evidence for a model including time, condition and time × condition (BF01 = 1/BF10). Values > 3 indicate strong evidence and are highlighted in bold

**Fig. 5:**
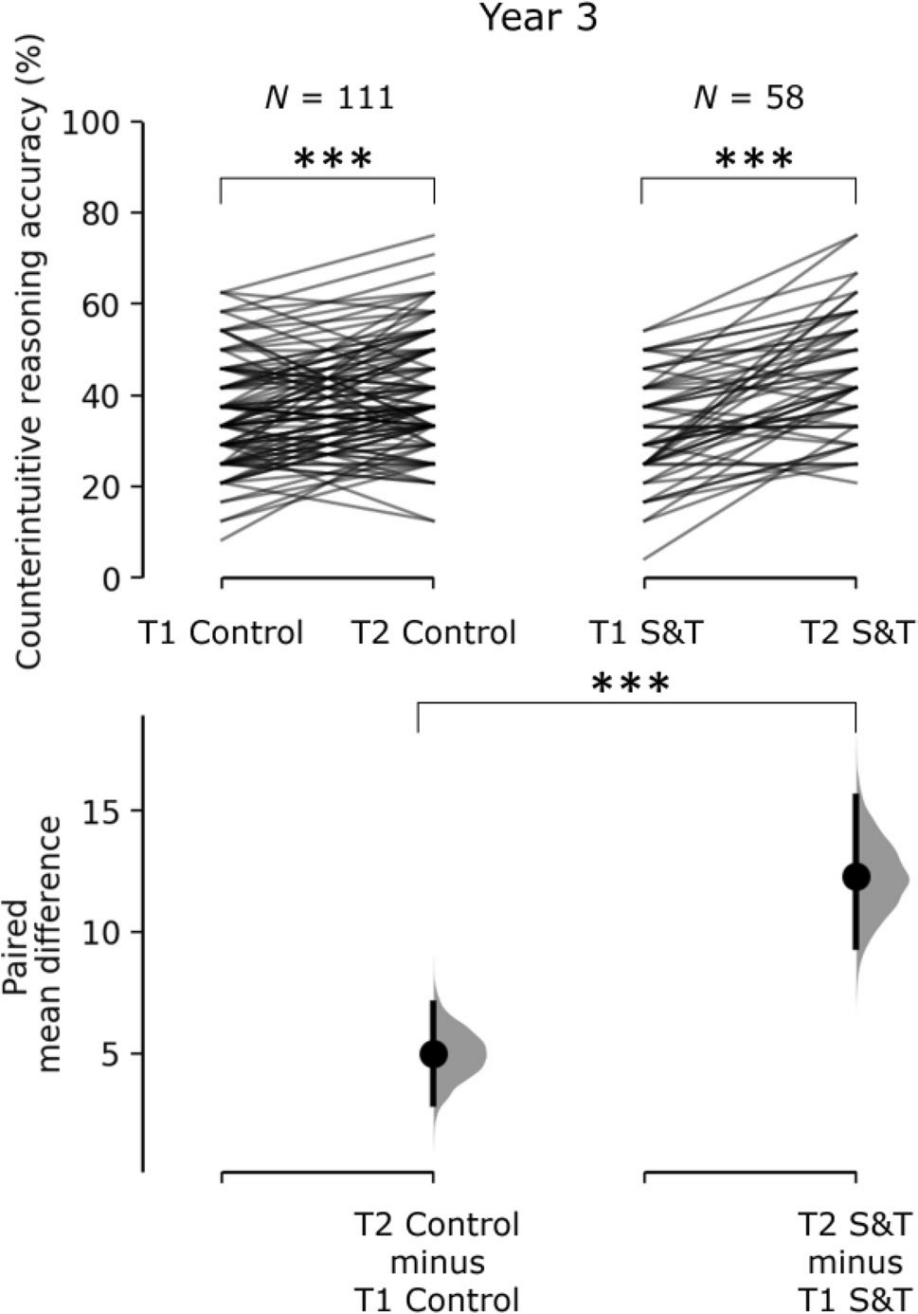
Cumming estimation plots of the paired mean difference between conditions for Year 3 children’s counterintuitive reasoning task accuracy. The raw accuracy data is plotted on the upper axes; each paired set of observations is connected by a line. On the lower axes, each paired mean difference is plotted as a bootstrap sampling distribution. Mean differences are depicted as dots; 95% confidence intervals are indicated by the ends of the vertical error bars. This figure was created using https://www.estimationstats.com/ (Ho et al., 2019). ^†^*p* ≤ 0.10, *p ≤ 0.05, **p ≤ 0.01, ***p ≤ 0.001

For Year 5 children, there was no significant time by condition interaction for any of the four measures, suggesting no specific improvements at a group level associated with the S&T intervention. For Year 3 children, there was a significant time by condition interaction for counterintuitive reasoning accuracy. Follow-up simple main effects showed that there was significant improvement in counterintuitive reasoning accuracy from T1 to T2 in both groups but the improvement was greater in the S&T group, *F*(1, 57) = 60.5, *p* < 0.001, η_p_^2^ = 0.515, than in the Control group, *F*(1, 110) = 22.2, *p* < 0.001, η_p_^2^ = 0.168 (Fig. 5). Follow-up analyses separating the active control and teaching as usual groups indicated that S&T led to greater improvements in counterintuitive reasoning accuracy in Year 3 than both control groups (Supplementary Materials D, Table S5, and Fig. S1).

Bayesian analyses indicate that there is strong evidence for a time × condition interaction for counterintuitive reasoning accuracy in Year 3 only. However, there was strong evidence for the null hypothesis, i.e. no interaction effect, in Year 5. Additional analysis using estimation of the paired mean T1 – T2 differences for Year 3 and Year 5 Stop & Think groups (https://www.estimationstats.com/, Ho et al., 2019) indicated that the intervention effect on counterintuitive reasoning accuracy was greater for Year 3 (mean difference = 12.3% [95%CI 9.3, 15.5]) than Year 5 children (mean difference = 5.5% [95%CI 2.9, 8.1]), with non-overlapping confidence intervals. For the executive function measures, there was strong evidence for the null hypothesis for all EF measures in both Year groups, except verbal WM in Year 5 (Table 5).

#### Predictors of Intervention Effects

Multiple regressions were run to assess predictors of T2 counterintuitive reasoning accuracy, in particular whether any predictor may specifically explain variance in accuracy changes in the Stop & Think group compared to the control group. In a first step, age at T2, T1 counterintuitive reasoning accuracy, the three EF measures and condition were entered as predictors. In a second step, stepwise entry was used to assess whether the interactions between any of four variables and condition explained additional variance in T2 counterintuitive reasoning accuracy. No interaction term was found to be significant. The step 1 models explained 37.1% variance in Year 3 (*F*(6, 144) = 14.1, *p* < 0.001) and 54.8% variance in Year 5 (*F*(6, 149) = 30.1, *p* < 0.001), with T1 counterintuitive reasoning accuracy a significant predictor of T2 counterintuitive reasoning accuracy in both Year groups, condition a significant predictor in Year 3 only, and WM a trend significant predictor in Year 5 only, in line with the mixed ANOVAs reported above (Table 7).

**Table 7:** Multiple regression testing for executive function predictors of T2 counterintuitive reasoning accuracy

Variables	Year 3			Year 5		
	β	*t*	*p*	β	*t*	*p*
T2 age	0.029	0.4	0.679	− 0.014	0.2	0.816
T1 counterintuitive reasoning accuracy	**0.543**	**8.1**	** < 0.001**	**0.653**	**11.1**	** < 0.001**
T1 inhibitory control	0.067	0.9	0.356	0.077	1.3	0.199
T1 verbal working memory	0.109	1.6	0.116	0.116	1.9	0.061
T1 visuospatial working memory	0.017	0.2	0.809	0.069	1.2	0.247
Condition^a^	**0.198**	**2.8**	**0.005**	0.017	0.3	0.778

^a^Condition is coded as 0: Teach as Usual and See + combined, 1: Stop & Think. Significant predictors are highlighted in bold

The analyses were then repeated with IQ as an additional variable. In Year 3, again no interaction term was found to be significant. The model explained 39.9% of variance (*F*(7, 141) = 13.4, *p* < 0.001), IQ was a significant regressor (Table 8). In Year 5, IQ was also a significant regressor and the interaction term between condition and IQ was found to be significant and entered stepwise in a second block. The final model explained 56.9% variance (*F*(8, 146) = 24.1, *p* < 0.001) (Table 8). Running separate multiple regressions for Control and S&T conditions, including the same variables except for condition and Condition × IQ to follow-up this interaction revealed that higher IQ predicted higher T2 counterintuitive reasoning accuracy in the Control condition (β = 0.244, *t* = 2.4, *p* = 0.021) but not in the S&T condition (β = 0.104, *t* = 1.0, *p* = 0.312).

**Table 8:** Multiple regression testing for executive function and IQ as predictors of T2 counterintuitive reasoning accuracy

Variables	Year 3			Year 5		
	β	*t*	*p*	β	*t*	*p*
T2 age	0.076	1.1	0.295	0.001	< 0.1	0.991
T1 counterintuitive reasoning acc	**0.466**	**6.5**	** < 0.001**	**0.560**	**8.2**	** < 0.001**
T1 inhibitory control	0.057	0.9	0.433	0.086	1.4	0.157
T1 verbal working memory	0.062	0.9	0.380	0.096	1.6	0.118
T1 visuospatial working memory	-0.025	0.4	0.719	0.038	0.6	0.520
IQ	**0.224**	**2.9**	**0.004**	**0.325**	**3.0**	**0.003**
Condition^a^	**0.192**	**2.8**	**0.006**	0.011	0.2	0.849
Condition^a^ × IQ				** − 0.205**	** − 2.1**	**0.035**

^a^Condition is coded as 0: Teach as Usual and See + combined, 1: Stop & Think

In summary, neither individual differences in EF nor IQ were found to modulate how much positive benefit children obtain from the S&T intervention.

## Discussion

Evaluations of EF training interventions have typically demonstrated improved performance on the trained EF task but have shown poor transfer to ‘real-world’ academic attainment (Berkman et al., 2014; Diamond & Lee, 2011; Diamond & Ling, 2016; Jacob & Parkinson, 2015; Serpell & Esposito, 2016). In contrast to this, a recent large-scale RCT (Palak et al., 2019) including 6672 Year 3 (7- to 8-year-olds) and Year 5 (9- to 10-year-olds) children demonstrated the effectiveness of subject-embedded IC training on improving children’s performance on standardised academic (mathematics and science) achievement tests. The intervention was based on the idea that IC is needed to inhibit prepotent naive beliefs that interfere with the application of valid conceptual knowledge in mathematics and science. In the current manuscript, we investigated the possible mechanisms by which this intervention works. First, using cross-sectional analyses, we hypothesised that children’s EF (in particular IC) would be positively associated with science and mathematics counterintuitive reasoning. Second, we predicted that children in S&T would show improved performance on a science and mathematics task focusing on counterintuitive concepts of the primary school curriculum compared to children partaking in a socio-emotional active control intervention (See +) or teaching as usual, and that they may also show improvement in a standard IC task, but not in other EF measures. Finally, we explored whether individual differences in EF or IQ predicted which children benefitted most from S&T training.

### Cross-sectional Associations Between Counterintuitive Reasoning and Executive Functions

A positive cross-sectional association was found (pre-training) between all EF measures and IQ and accuracy in the counterintuitive reasoning task in Year 5 only. Verbal WM was the only unique EF predictor of counterintuitive reasoning accuracy and remained a significant predictor when controlling for age and IQ. In Year 3, IQ, but not EF, was correlated with counterintuitive reasoning accuracy. Statistical comparisons indicated that the relationship between counterintuitive reasoning accuracy and IQ and verbal WM was significantly greater in Year 5 than Year 3. While WM has been at the forefront of many studies investigating the relationship between EF and achievement (Friso-van den Bos et al., 2013), research examining counterintuitive reasoning has predominantly focused on IC (e.g. Brault Foisy et al., 2015; Brookman-Byrne et al., 2019; Kwon & Lawson, 2000; Masson et al., 2014). By extending the literature to examine verbal and visuospatial WM in addition to IC, we have provided evidence to suggest that WM also contributes to counterintuitive reasoning. The lack of specific correlation between IC and counterintuitive reasoning when controlling for age and other EFs challenges the hypothesis that children need to inhibit their intuitive beliefs to correctly reason about counterintuitive concepts (Mareschal, 2016; Vosniadou et al., 2018). Instead, the fact that all three measures of EF correlated with counterintuitive reasoning accuracy in Year 5 suggests that a general EF factor is important for counterintuitive reasoning and that future studies should incorporate a wide array of EF measures when investigating predictors of counterintuitive reasoning. Previous evidence for a unique role of verbal WM (compared to visuospatial WM or IC) in mathematics conceptual reasoning has been found in children and adults (Cragg et al., 2017). Here we extend this work by showing that verbal WM may also play a specific role in the understanding of *counterintuitive* concepts^1^. However, one limitation to be aware of is that the association we observed could be due to the use of numerical stimuli in the verbal WM task—indeed mathematics and science problems often involve numbers and this could have inflated the correlation observed in this study.

IC is recognised as a difficult construct to measure, and there are many different types of IC (Nigg, 2000). The lack of specific associations between IC and counterintuitive reasoning in Years 3 and 5 may be because the aspect of IC required to accurately solve counterintuitive reasoning problems is not the same as the response inhibition required by our computerised whack-a-mole task (Green et al., 2019). Indeed, Brookman-Byrne et al. (2018) found that interference control (and not response inhibition as measured by the whack-a-mole task) was associated with better accuracy scores on a mathematics and science counterintuitive reasoning task in young adolescents. Thus, it remains possible that the lack of associations observed in this study reflect the choice of IC task and the selection of a task requiring interference control aspects of IC would have revealed the predicted association.

While we expected that both Year groups would show associations between EF measures and counterintuitive reasoning, no association was observed in Year 3, and the association between verbal WM and counterintuitive reasoning was significantly greater in Year 5 than Year 3. Developmental differences in the association between EF and academic achievement have been documented previously. For example, greater associations have been found between IC and mathematics in younger children (Blair & Razza, 2007; Espy et al., 2004) than older children (Bull & Scerif, 2001; Brookman-Byrne et al., 2018; Cragg et al, 2017; Donati et al., 2019; Szűcs et al., 2013, 2014), while associations between WM and mathematics appear to be more stable across ages (Cragg et al., 2017; Donati et al., 2019; Dumontheil & Klingberg, 2012; Friso-van den Bos et al., 2013; Lee & Bull, 2015). However, the Year group differences found here do not reflect the changes observed in the literature, as they would suggest a greater association between IC and counterintuitive reasoning in the younger children. Our measures may have been somewhat less reliable in the younger children (T1-T2 correlation of the counterintuitive reasoning measure was significantly lower for Year 3 children than Year 5 children), which could have limited our ability to observe significant cross-sectional associations, but these differences are unlikely to have fully accounted for the age differences in association observed. Furthermore, the content of the counterintuitive reasoning assessments for the different year groups differed, in line with the concepts covered in the curriculum. It is therefore possible that the items included in the Year 5 assessment drew on the variables measured by our EF tasks, particularly verbal WM, more than the items included in the Year 3 assessment.

It is important to acknowledge that beyond the core IC, WM and shifting EFs, both mathematics and science have been found to be associated with skills such as spatial scaling, mental transformation and planning in children (Gilligan et al., 2019; Hodgkiss et al., 2018; Mayer et al., 2014) and with analogical reasoning in adolescents (Brookman-Byrne et al., 2019).

### Stop & Think Intervention Effects on Counterintuitive Reasoning

As predicted, there was a positive effect of the Stop & Think intervention on counterintuitive reasoning, although for Year 3 children only. There was a significant intervention effect for Year 3 counterintuitive reasoning accuracy compared to the Control group (TAU and See + combined) as well as compared to the active control (See +) alone and the TAU control condition alone. Importantly, the beneficial effects of the Stop & Think intervention were observed even though the children in the S&T conditions were not provided with additional content or exposure time to mathematics and science content (the S&T intervention took place within a scheduled mathematics or science lesson). These findings support our hypothesis that EF training that is embedded within the specific domain in which it is to be applied (i.e. science and mathematics content from the school curricula) can improve children’s performance on English National Curricula-based tasks, and specifically, that training children to ‘stop and think’ can help them accurately respond to counterintuitive science and mathematics problems.

No effect of the intervention was observed for Year 5 children, and the Stop & Think intervention effect was also found to be significantly greater in year 3 than Year 5 children. While the mathematics and science counterintuitive reasoning test was slightly easier for Year 5 than Year 3 children, there was no ceiling effect which could have masked the impact of the S&T intervention. The lack of benefit from the intervention for this Year group is not consistent with the effects of the full RCT sample, in which Year 5 children demonstrated a positive benefit from the intervention, on mathematics and science, whilst the Year 3 children did not (with the exception of those on free school meals, who benefitted for mathematics only). It is, however, consistent with data from *N* = 456 children who took part in a pilot version of Stop and Think (Wilkinson et al., 2020), where it was found that Year 3 children, but not Year 5 children, demonstrated improved counterintuitive reasoning following the Stop and Think intervention, compared to a TAU control. To reconcile these findings, it is important to understand that, while both the current study and the pilot study used accuracy on a computerised counterintuitive mathematics and science reasoning task as their primary measure of the effectiveness of the intervention, the RCT study (Palak et al., 2019) used performance on standardised pen-and-paper academic mathematics and science tests as their primary measure of effectiveness. Thus, it appears that for the younger children in Year 3, the training with domain embedded IC is limited to benefits on the performance of a computerised reasoning tasks testing the same or similarly framed counterintuitive problems as the intervention itself. Instead, for older Year 5 children, the same training leads to benefits on performance of general mathematics and science standardised tests, but not specifically on counterintuitive problems.

The current study showed greater association between EF measures (verbal WM specifically) and counterintuitive reasoning in Year 5 children. The RCT reported greater transfer of the Stop & Think intervention to standardised mathematics and science test in Year 5 children, while no specific improvements in counterintuitive reasoning were found in the present study. Put together, these results could suggest that the older children are more capable of building on their greater EF abilities to incorporate the stop-and-think strategy broadly across a range of context and problem types. This interpretation fits with work showing that children with higher WM and mathematics skills at baseline respectively benefitted more from a WM and number line training programmes (Nemmi et al., 2016). The fact that we did not observe improvements on the counterintuitive reasoning test in Year 5 in the present study could be due to the limited number of trials of the test, which meant, with the current sample size, it was not sufficient to detect an effect (compared to the larger number of problems used in 1-h-long standardised tests).

We also predicted that training IC within science and mathematics might lead to improvements in IC in other domains but no evidence of this was observed in either age group. This suggests that the impact of subject-embedded IC training does not transfer to lab-based EF measures. This finding seems reasonable given previous research that lab-based EF training does not transfer to everyday EF (Diamond & Ling, 2016). It then follows that the opposite would also be true, i.e. that subject-embedded EF training would not transfer to lab-based EF tasks performance.

### Predictors of Counterintuitive Reasoning

The final aim of this study was to further investigate potential mechanisms through which the Stop & Think intervention may benefit science and mathematics performance. It has been argued that considering the role of individual differences in the responsiveness to training is important to try to develop better training interventions (Smid et al., 2020). To do so, exploratory analyses tested whether characteristics of participants at baseline influenced how much they benefited from S&T over a few months, compared to the other conditions. On the one hand, S&T could benefit children with low IC skills pre-training by encouraging practice of inhibiting a dominant response and waiting until making a response. On the other, a certain level of maturation of the neural systems supporting IC skills pre-training may be needed for children to then practice applying IC in the context of mathematics and science counterintuitive reasoning. In fact, individual differences in executive function were not found to predict variance in the effectiveness of the Stop & Think intervention. This null finding is likely driven by the fact that a beneficial effect of S&T on counterintuitive mathematics and science accuracy was only found in Year 3 children, who showed less associations between counterintuitive reasoning and EF measures to start with. Future work should investigate whether individual differences in other aspects of IC, or other aspects of cognition, better predict individual differences in the benefit of S&T.

## Conclusion

The aim of this study was to investigate the role of executive functions in counterintuitive mathematics and science counterintuitive reasoning as a putative cause for the effectiveness of the S&T intervention. We found cross-sectional associations between counterintuitive reasoning and EF in children aged 9 to 10 years (Year 5), but not children aged 7 to 8 years (Year 3), with evidence of a specific role of verbal WM. A domain-dependent IC training intervention benefited counterintuitive reasoning in 7- to 8-year-olds only, replicating previous findings. EF measures did not predict which children would most benefit from the intervention. A previous RCT had found Year 5 children, but not Year 3 children, showed improvements in standardised mathematics and science attainment measures. Combined, these results suggest that individual differences in EF play a lesser role in counterintuitive reasoning in younger children, who show near transfer of the training on counterintuitive science and mathematics problems, while older children show a greater association between EFs and counterintuitive reasoning and are able to apply the strategies developed during the S&T training to mathematics and science subjects more broadly. While developing interventions that will improve academic performance is invaluable to society, understanding how and why these interventions work is critical to improving the interventions and developing future interventions (Thomas et al., 2019) .

### Supplementary Information

Below is the link to the electronic supplementary material.

Supplementary file1 (DOCX 167 kb)

## Data Availability

The data that support the findings of this study are available from the corresponding author upon request.

## Footnotes

1 Interestingly the results also revealed a trend longitudinal association between verbal WM at T1 and counterintuitive reasoning accuracy at T2, when controlling for T1 accuracy. This result will need to be replicated but provides further support for a specific role of verbal WM in counterintuitive reasoning.
