# Supplementary material for: How Do Executive Functions Influence Children’s Reasoning About Counterintuitive Concepts in Mathematics and Science?
Source: J Cogn Enhanc. 2023 Sep 21;7(3-4):257–75. doi: 10.1007/s41465-023-00271-0 (PMC10770252; doi:10.1007/s41465-023-00271-0)
Supplement: Supplementary file 1 — Supplementary file1 (DOCX 167 kb) [file 41465_2023_271_MOESM1_ESM.docx]

**Supplementary Materials:**

**How do executive functions influence children’s reasoning about counterintuitive concepts in mathematics and science?**

**A. Time 1 cross-sectional analyses of executive function scores for Year 3 and Year 5 children**

Independent *t*-tests indicated that Year 5 children had higher scores on the two working memory (WM) tasks and the inhibitory control (IC) task *d*’ measure (large effect sizes) than the Year 3 children, while neither IQ nor the IQ sub-tests scores (which are corrected for age) differed between the Year groups (**Table S1**).

**Table S1:** Descriptive statistics and Year group comparisons of the key Time 1 measures.

|  | **Year 3** | | | **Year 5** | | | **Year groups comparison** |
| --- | --- | --- | --- | --- | --- | --- | --- |
|  | ***N*** | ***M*** | ***SD*** | ***N*** | ***M*** | ***SD*** |  |
| **IQ^a^** | 184 | 102.3 | 14.7 | 180 | 103.3 | 14.1 | *t*(362) = 0.66, *p* = .508, *d* = 0.07 |
| **Vocabulary** WASI-II Vocabulary subtest t-score | 184 | 54.4 | 9.5 | 181 | 54.3 | 8.5 | *t*(363) = 0.06, *p* = .954, *d* = 0.01 |
| **Matrix reasoning** WASI-II Matrix Reasoning subtest t-score | 185 | 48.2 | 10.6 | 181 | 49.5 | 10.5 | *t*(364) = 1.10, *p* = .270, *d* = 0.12 |
| **Verbal working memory**  backwards digit score | 169 | 4.28 | 2.22 | 170 | 6.51 | 2.69 | *t*(337) = 8.34, *p* < .001, *d* = 0.90 |
| **Visuospatial working memory** follow Frankie score | 187 | 9.04 | 3.68 | 182 | 11.87 | 3.53 | *t*(367) = 7.54, *p* < .001, *d* = 0.79 |
| **Inhibitory control** whack-a-mole *d*-prime  **whack-a-mole go RT (ms)** | 185  185 | 1.96  284 | 0.67  41 | 180  180 | 2.58  270 | 0.73  39 | *t*(363) = 8.54, *p* < .001, *d* = 0.89  *t*(363) = 3.36, *p* < .001, *d* = 0.11 |
| **Counterintuitive reasoning accuracy (%)** | 183 | 34.2 | 11.5 | 183 | 44.6 | 15.7 |  |

*^a^ IQ is a combination of the Vocabulary and Matrix Reasoning sub-tests of the WASI II (Wechsler, 2011)*

**B. Cross-sectional analyses considering vocabulary and matrix reasoning measures separately**

While this was not the main focus of the paper, partial correlations (**Table S2**) and multiple regression (**Table S3**) assessing associations between IQ, executive function (EF) measures and counterintuitive reasoning performance were repeated using the separate vocabulary and matrix reasoning measures of the WASI II (Wechsler, 2011). Results are consistent with those presented in the main paper with regards to associations with EFs. Vocabulary and matrix reasoning were both found to correlate with counterintuitive reasoning accuracy (**Table S2**) and the measures explained unique variance when entered in the multiple regression (**Table S3**). In the multiple regression, as in the analyses presented in the main text, counterintuitive reasoning accuracy was entered as the dependent variable, age (model A), or age and IQ measures (model B) were entered as control variables in step 1 and the unique variance explained by the executive function measures was then assessed in step 2.

**Table S2:** Partial parametric correlations between vocabulary and matrix reasoning measures, and Time 1 (T1) executive function measures and mathematics and science counterintuitive reasoning accuracy, covarying age. N’s for each test are provided below the diagonal.

| **Year** | **Variables** | **1.** | **2.** | **3.** | **4.** | **5.** | **6.** | **7.** |
| --- | --- | --- | --- | --- | --- | --- | --- | --- |
| **Year 3** | **1. Vocabulary** |  | .428*** | .394*** | .200** | .118 | .019 | .386*** |
|  | **2. Matrix reasoning** | 181 |  | .114 | .339*** | .158* | -.035 | .280*** |
|  | **3. T1 verbal working memory** | 163 | 164 |  | .226** | .124 | -.003 | .064 |
|  | **4. T1 visuospatial working memory** | 181 | 182 | 166 |  | .188* | -.027 | .120 |
|  | **5. T1 inhibitory control *d*’** | 179 | 180 | 164 | 182 |  | .057 | .076 |
|  | **6. T1 inhibitory control go RT** | 181 | 182 | 166 | 184 | 182 |  | .041 |
|  | **7. T1 counterintuitive reasoning accuracy** | 177 | 178 | 162 | 180 | 178 | 180 |  |
| **Year 5** | **1. Vocabulary** |  | .475*** | .205** | .263*** | .106 | -.068 | .537*** |
|  | **2. Matrix reasoning** | 177 |  | .353*** | .364*** | .290*** | -.008 | .553*** |
|  | **3. T1 verbal working memory** | 166 | 167 |  | .285*** | .226** | -.008 | .345*** |
|  | **4. T1 visuospatial working memory** | 178 | 178 | 167 |  | .296*** | -.023 | .256*** |
|  | **5. T1 inhibitory control *d*’** | 176 | 176 | 165 | 177 |  | -.175* | .179* |
|  | **6. T1 inhibitory control go RT** | 176 | 176 | 165 | 177 | 177 |  | .068 |
|  | **7. T1 counterintuitive reasoning accuracy** | 178 | 178 | 167 | 179 | 177 | 177 |  |

*Note*. *p* ≤ .10, * *p* ≤ .05, ** *p* ≤ .01, *** *p* ≤ .001

**Table S3:** Follow-up multiple regression analysis of Year 5 data, including vocabulary and matrix reasoning measures separately.

|  |  | **Model A** | | **Model B** | |
| --- | --- | --- | --- | --- | --- |
|  |  | **β** | ***p*** | **β** | ***p*** |
| **(Step 1)** | **T1 age** | .046 | .619 | .059 | .360 |
|  | **Vocabulary** |  |  | **.289** | **<.001** |
|  | **Matrix reasoning** |  |  | **.346** | **<.001** |
| **(Step 2)** | **T1 verbal WM** | **.274** | **<.001** | **.153** | **.026** |
|  | **T1 visuospatial WM** | .104 | .181 | -.026 | .708 |
|  | **T1 inhibitory control** | .108 | .165 | .032 | .637 |
|  |  | Step 1: *R*^2^ = 0.2%, *n.s.*  **Step 2:** **Δ*R*^2^ = 13.2%, *p* < .001** | | **Step 1: *R*^2^ = 35.2%, *p* < .001**  Step 2: Δ*R*^2^ = 2.2%, *n.s.* | |

*Note.* Parameter estimates and *p*-values are provided for the final models. Significant effects are highlighted in bold. WM: working memory.

Fisher r-to-z transformation comparison of correlations between Year groups showed significantly greater correlation in Year 5 than Year 3 between vocabulary and verbal WM (*Z*_Y3vsY5_ = -2.293, *p* = .022) and counterintuitive reasoning accuracy at T1 (*Z*_Y3vsY5_ = -3.134, *p* = .002), but no other significant Year group differences (all other |*Z|*’s < 1.874, *p’*s > .06).

**C. Gender, age and IQ intervention conditions comparisons**

Chi-square tests showed that S&T and Control groups did not differ in gender distribution (Year 3: Χ^2^(1) = 2.40, *p* = .121; Year 5: Χ^2^(1) = 2.36, *p* = .124) (**Table 1**). The two groups did not differ in terms of IQ either (Year 3: *F*(1, 165) = 0.91, *p* = .342; Year 5: *F*(1, 166) = 1.04, *p* = .310). Time (T1, T2) x Condition (Control, S&T) mixed repeated measures ANOVAs were conducted to compare age between conditions. In Year 3 children in the S&T condition were on average older than children in the Control condition across time points (*F*(1, 167) = 7.06, *p* = .009; Control: *M* = 7.99 years (*SE* = 0.03); S&T: 8.13 years (0.04)). The Time x Condition interaction was trending to significance (*F*(1, 167) = 3.39, *p* = .067). In Year 5 children in the S&T condition were on average younger than in children in the Control condition across time points (*F*(1, 168) = 18.11, *p* < .001; Control: 10.19 (0.04); S&T: 9.97 (0.04)). In addition, there was a significant Time x Condition interaction (*F*(1, 168) = 8.82, *p* < .001), with greater increase in age in the S&T condition (T1: *M* = 9.80 years (*SD* = 0.35); T2: 10.14 (0.33)) than in the Control condition (T1: 10.03 (0.33), T2: 10.35 (0.32)). As the main intervention effect finding was found in Year 3 children and was significant when age at T2 was entered in the multiple regression analyses, these age differences are not a problematic confound. However, they may have contributed to the lack of strong evidence for either hypothesis on some measures in the Year 5 children.

**D. Analyses separating the teaching as usual and active control See + group**

Main analyses combined the teaching as usual (TAU) and active control group (See+), as was done in the main UnLocke RCT (Palak et al., 2019). Here descriptives of these two control groups are provided in **Table S5**. In addition, analyses comparing intervention effects on counterintuitive reasoning performance between the three conditions (TAU, See+ and Stop & Think) are reported in **Table S6**.

**Table S4:** Descriptive statistics of the samples included in the analyses of effects of the Stop & Think (S&T) intervention splitting the Control group into See+ and TAU.

|  |  | **Science and mathematics counterintuitive reasoning** | | | |
| --- | --- | --- | --- | --- | --- |
|  |  | ***N*** | **Age at T2 *M* (*SD*)** | **% males** | **IQ *M* (*SD*)** |
| **Year 3** | **S&T** | 58 | 8.28 (0.29) | 62.1 | 100.9 (15.4)^a^ |
|  | **See+** | 73 | 8.15 (0.31) | 50.7 | 101.7 (14.5)^a^ |
|  | **TAU** | 38 | 8.15 (0.34) | 47.4 | 106.2 (14.6) |
| **Year 5** | **S&T** | 93 | 10.14 (0.33) | 62.4 | 104.2 (15.6)^a^ |
|  | **See+** | 35 | 10.30 (0.32) | 48.6 | 102.7 (14.8) |
|  | **TAU** | 42 | 10.38 (0.33) | 52.4 | 101.3 (9.5)^a^ |

*Note.* The S&T group descriptive statistics are repeated here to facilitate comparison.

^a^ One IQ value was missing for this group.

Mixed repeated measures ANOVAs comparing the three conditions pairwise were performed. The results showed that the interaction between time and condition observed when the control groups were combined for the counterintuitive reasoning accuracy measure was also significant when Stop & Think was compared separately to the TAU and See+ control groups (**Table S6**, **Figure S1**). Follow-up paired t-tests indicated that the effect of time was significant in all three conditions, but larger in Stop & Think (*t*(57) = 7.8, *p* < .001) than in See+ (*t*(72) = 4.4, *p* < .001) and TAU (*t*(37) = 2.0, *p* = .048) (**Figure S1**).

**Table S5:** Results of follow-up 2 (Time: Time 1, Time 2) x 2 (Condition: Stop & Think, Control) mixed ANOVAs carried out on counterintuitive reasoning accuracy for Year 3 children contrasting three intervention conditions.

|  |  | **Main effect of time** | | | **Main effect of group** | | | **Time x group interaction** | | |
| --- | --- | --- | --- | --- | --- | --- | --- | --- | --- | --- |
|  | df | *F* | *p* | η_p_^2^ | *F* | *p* | η_p_^2^ | *F* | *p* | η_p_^2^ |
| **S&T vs See+** | **1, 129** | **80.4** | **< .001** | **.384** | 1.4 | .238 | .011 | **11.8** | **< .001** | **.084** |
| **S&T vs TAU** | **1, 94** | **41.8** | **< .001** | **.308** | 1.1 | .295 | .012 | **10.6** | **.002** | **.101** |
| **See+ vs TAU** | **1, 109** | **18.1** | **< .001** | **.143** | **4.8** | **.031** | **.042** | 0.4 | .527 | .004 |

*Note.* Significant effects are highlighted in bold. S&T: Stop and Think; See+: socio-emotional intervention; TAU: teaching as usual.


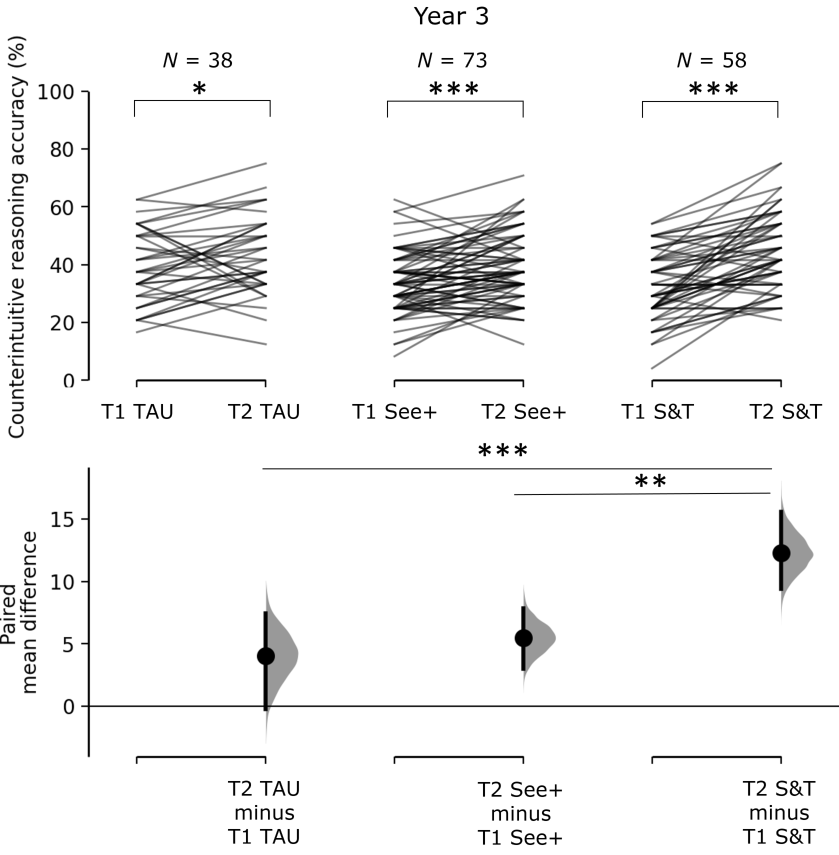


**Figure S1.** Cumming estimation plots of the paired mean difference between conditions for Year 3 children’s counterintuitive reasoning task accuracy. The raw accuracy data is plotted on the upper axes; each paired set of observations is connected by a line. On the lower axes, each paired mean difference is plotted as a bootstrap sampling distribution. Mean differences are depicted as dots; 95% confidence intervals are indicated by the ends of the vertical error bars. This figure was created using <https://www.estimationstats.com/> (Ho et al., 2019).  ^†^ *p* ≤ .10, * *p* ≤ .05, ** *p* ≤ .01, *** *p* ≤ .001

**References**

Ho, J., Tumkaya, T., Aryal, S., Choi, H., & Claridge-Chang, A. (2019) Moving beyond P values: Everyday data analysis with estimation plots. *Nature Methods*, *16*(7), 1548-7105.

Palak, R., Rutt, S., Easton, C., Sims, D., Bradshaw, S. & McNamara, S. (2019, September). Stop and Think: Learning Counterintuitive Concepts Evaluation Report. Retrieved from <https://educationendowmentfoundation.org.uk/projects-and-evaluation/projects/learning-counterintuitive-concepts/>

Wechsler, D. (2011) *Wechsler abbreviated scale of intelligence (WASI-II)*. (2nd ed.). San Antonio, TX: Pearson.
